# Preoperative intravenous iron treatment – a cohort study on colorectal cancer recurrence

**DOI:** 10.1016/j.sopen.2023.09.003

**Published:** 2023-09-17

**Authors:** Magnus Ploug, Niels Qvist, Rasmus Kroijer, Torben Knudsen

**Affiliations:** a Department of Regional Health Research, University of Southern Denmark, Denmark; b Department of Surgical Gastroenterology, Hospital South West Jutland, Region of Southern Denmark, Denmark; c Research Unit for Surgery, Odense University Hospital, Odense, Denmark; d University of Southern Denmark, Odense, Denmark; e Department of Medical Gastroenterology, Hospital South West Jutland, Region of Southern Denmark, Denmark

**Keywords:** Iron deficiency, anemia, iron treatment, Colorectal, cancer, Surgery, Recurrence

## Abstract

**Background:**

Intravenous (i.v.) iron treatment has been speculated to increase the malignant potential of colorectal malignancies but also to enhance the immune systems potential to fight the invasive tumor. Clinical data however is very limited. We investigate if preoperative i.v. iron treatment is associated with colorectal cancer (CRC) recurrence.

**Methods:**

Retrospective cohort study on surgical CRC patients with iron deficiency anemia (1st March 2013 - 31st December 2019). Patients were grouped based on whether they had received preoperative treatment with i.v. iron. Local data was combined with data from the National Danish Health registries to identify recurrences, death, and emigration. Survival analysis, including Kaplan-Meyer curves and multivariate competing risk analysis adjusting for sex, age, ASA-group, tumor stage, surgical radicality, and miss match repair status was performed.

**Results:**

Of 1228 patients, 125 were available for analysis. 89 patients had received preoperative i.v. iron and 36 had not. The two groups were comparable on baseline and surgical characteristics. Median follow-up times were 4.74 in iron treated patients and 5 years in patients not receiving iron treatment. Five-year rate of non-recurrence was 85 % (0.74–0.91) in the i.v. iron treated group vs. 82 % (0.64–0.91) in the control group, non-significant difference. Multivariate survival analysis did not find iron treatment to be associated with recurrence rates (Hazard Ratio 0.88 (95 % ci; 0.31–2.51).

**Conclusion:**

No association between preoperative i.v. iron treatment and the five-year cancer recurrence rate in iron deficient anemic CRC patients was found.

## Introduction

We have previously shown that 47 % of patients diagnosed with colorectal cancer (CRC) are anemic and that 64 % are iron deficient [1]. Preoperative anemia is associated with higher rates of postoperative complications and mortality after both elective colon surgery [2] and general (non-cardiac) surgery [3,4]. On this background, preoperative iron treatment is recommended by several clinical guidelines for the treatment for CRC [[5], [6], [7], [8]]. Randomized studies, however, have not found this intervention to lower complication or mortality rates following abdominal surgery [[9], [10], [11]].

The impact of anemia and iron deficiency on long-term oncological outcomes is not clear. In CRC patients, a meta-analysis found anemia associated with lower overall-survival (OS) and disease-free-survival (DFS) [12], while newer studies, find no association with DFS, and conflict on the association to OS [13,14]. When focusing on iron status both low and high levels of iron are associated with lower OS [15].

Theoretically, preoperative iron supplementation may exert both positive and negative influences on cancer recurrence. Iron is an essential micronutrient for gastrointestinal cancer development and growth [16,17]. Thus, the systemic inflammatory reaction often seen in colorectal cancer patients could be an innate defense mechanism against tumor growth by limiting the uptake and availability of iron [18]. On the other hand, iron is also crucial for the function of the immune system and thus iron deficiency could increase the metastatic potential of malignancies by impairing the immune system [19,20].

One randomized controlled trial, the IVICA trial, has published long-term oncological data, comparing preoperative oral with intravenous (i.v.) iron treatment, in anemic colorectal cancer patients [21]. The IVICA trial found no difference in OS or DFS. A matched retrospective cohort study on surgical CRC patients receiving either i.v. iron or no treatment also failed to find any difference on OS or DFS [22]. The rationale for preoperative iron treatment in the anemic patient without iron deficiency may be debatable, yet both studies were performed on anemic patients in general. Also, when reporting DFS, recurrence and death is combined as a composite endpoint [23]. Considering that most patients who die following curatively intended colorectal surgery die of other causes than CRC recurrence, it might be more meaningful to study recurrence alone [24]. We believe that this approach more accurately examines the oncological impact of iron treatment.

In this study on CRC patients, we therefore included only iron deficient anemic patients and chose recurrence as the primary endpoint when studying the association between preoperative i.v. iron treatment and long-term oncological outcome.

## Methods

This is a single-center retrospective cohort study on iron deficient anemic CRC patients. We compare CRC recurrence rates, using survival analysis, after intended curative surgical resection between patients who received preoperative i.v. iron with those who did not. Patients were identified from the Esbjerg Colorectal Hematological (ECH) cohort, described in detail elsewhere [1,25]. Preoperative i.v. iron treatment has been recommended at our institution for patients with iron deficiency anemia for the entire study period.

### Data sources

From the Danish Colorectal Cancer group (DCCG) database, we retrieved demographical, patient- and tumor-specific information as well as data on preoperative oncological treatment. The DCCG database includes adult patients diagnosed with their first colorectal adenocarcinoma, and has a validated completeness of 98.5 % [26]. Data was integrated with local laboratory data (hemoglobin (Hb), ferritin and transferrin saturation (TSAT)) and regional data on blood transfusions. Finally, we obtained data from four national Danish registries (Table A). Patients were linked across databases and registries using the Central Personal Registry number, a unique identification number assigned to Danish residents.

### Study population

Patients from the ECH-cohort diagnosed between 1st March 2013 and 31st December 2019, and registered in the DCCG database were identified. Surgical CRC patients were included if diagnosed with iron deficiency anemia upon CRC diagnosis. Excluded were those receiving non-curatively intended surgery, acute surgery, primary open or local endoscopic surgery, neoadjuvant oncological treatment, together with those who experienced a waiting period of >60 days from diagnosis to operation and those who had CRC surgery performed outside our facility. These criteria were the same as used previously on this cohort [25].

**Fig. 1:**
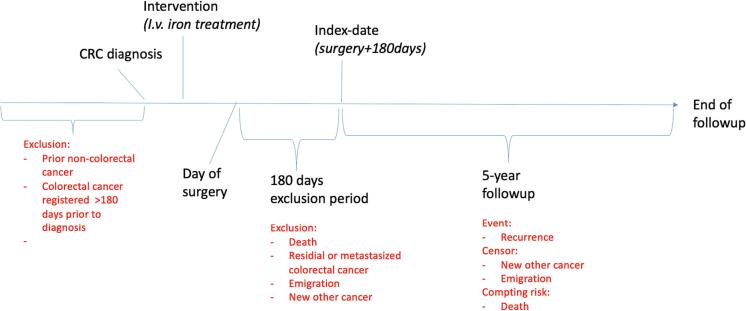
The time-flow and design of the study.

Based on registration in either The Danish National Patient Register (NPR) or the Danish Cancer Registry (DCR), we imposed additional exclusion criteria for the current study to ensure that the recurrences identified were indeed of CRC origin (Fig. 1). Based on the International Classification of Diseases, 10th Revision (ICD-10), we excluded patients with a prior malignant diagnosis (ICD-10 C00-C97) registered at any time before their CRC diagnosis. Exempted from this exclusion were non-melanoma skin cancers (NMSC) (ICD-10 C44) and the registration of CRC (ICD-10 C18-C20) <180 days before the diagnostic date of the current CRC. In addition, patients diagnosed with cancers other than CRC or NMSC within 180 days after the day of surgery were excluded. To avoid confusing actual recurrence with non-radical treatment of primary cancer we excluded patients with metastatic (ICD-10 C76-C80 or C991) or recurrent disease (C189X and C209X) registered within 180 after surgery. Finally, patients who died or emigrated within 180 days after surgery and patients not registered in the CPR-registry were excluded.

### Definition of variables

The primary outcome variable CRC recurrence was determined using a validated algorithm described previously [27]. Compared to the referenced algorithm we did one minor adjustment in including the specific ICD10 codes for local recurrence (C99) in the registration of recurrence. Those codes were introduced to the ICD10 nomenclature in 2018, thus after the original algorithm was established. Recurrence was defined as “(i) tumor growth at or near the site of the original tumor and in the same organ (colon or rectum) or (ii) metastases to tissue adjacent to the original tumor site or to a distant organ” [27] and diagnosed >180 days after colorectal surgery. In practice, recurrence was identified as either:

i) DNPR or DCR registered metastases code (ICD10 C76–C80) 180 or more days after colorectal cancer surgery.
ii) DNPR registered cytostatic therapy codes (BWHA1–2, BOHJ17, BOHJ19B1) 180 or more days after colorectal cancer surgery and 60 or more days after their last cytostatic therapy code.
iii) SNOMED-codes in DPR representing recurrence 180 or more days after colorectal cancer surgery. Combinations were “(i) T code (topography/location) in the colon or rectum (T6491, T65900, T65901, T65925, T65926, T660, T67 or T68) with morphology codes M8 or M9 with ≥ 3 in the fifth position (e.g., M8XXX3), (ii) any T code with morphology codes M8 or M9 with the numbers 4, 6 or 7 in the fifth position, (iii) liver adenocarcinoma (T56 in combination with M81403)” [27], assumed to represent a liver metastasis.
iv) DNPR codes of recurrence (ICD-10 C99) and codes specific of colorectal cancer recurrence (ICD-10 C189X and C209X).

Anemia was defined according to the World Health Organization criteria as male Hb <13.0 g/dl (8.1 mmol/l) and female Hb <12.0 g/dl (7.4 mmol/l) [28]. Iron deficiency was defined as a p-ferritin <50 μg/l [29,30]. Laboratory values taken closest to the day of diagnosis and prior to any i.v. iron treatment or blood transfusions constituted the baseline level. I.v. iron (ferric derisomaltose/iron isomaltoside 1000, MonoFer®, Pharmacosmos A/S, Denmark) was administered as a single dose according to a modified Ganzoni formula: I.v. iron dose (mg) = (body weight (kg) × (target Hb (g/dl) − actual Hb (g/dl)) × 2.4. + storage iron (set at 500 mg) where the target Hb was 15.1 g/dl in male and 13.5 g/dl in female [31]. The maximal dose to be administered was 20 mg/kg. Previous study on the ECH cohort has shown a median time from i.v. iron administration to surgery of nine days [25].

### Statistics

Statistical analyses were performed using Stata/IC 16.0 (Stata Corp, Texas, USA). Statistical significance was taken at p <0.05. Non-parametric data was reported as medians with interquartile ranges and compared using Wilcoxon rank-sum test while parametric data was reported as means with standard deviations and compared using student *t*-test. Categorical data was reported as percentages and compared using chi-squared test or in case of less than five individuals in a group by Fishers Exact test.

For time-to-event analysis, the day of surgery +180 days was the index date - the time follow-up began. Recurrence as defined above was the primary event of interest. Patients were censored from the day of emigration or date of diagnosis with non-colorectal cancer (other than NMSC) (Fig. 1). Death was considered a competing risk. Follow-up ended on date of recurrence, death, censoring, or at the end of five-year follow-up – which ever came first.

Time-to-recurrence is presented using Kaplan-Meyer curves. Subdistribution hazard ratios were computed using multivariate competing risk analysis and presented with 95 % confidence intervals (complete case analysis). The multivariate model includes i.v. iron treatment (yes/no) as the exposure variable. The independent variables were those most likely to affect recurrence on their own, thus potentially confounding our results and included age (continuous), gender (dichotomous), UICC tumor stage (dichotomized in UICC 1–2 and UICC 3–4), perivascular invasion (dichotomous), ASA-classification (dichotomized in ASA 1–2 and ASA > 2) and miss match repair status (dichotomous). The log transformed cumulated time-to-recurrence curves on i.v. iron were visually inspected to assess the proportional hazards assumption.

## Results

**Fig. 2:**
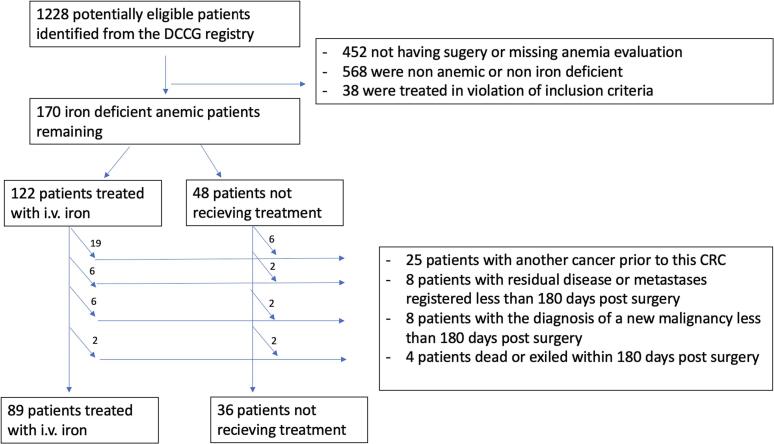
Flow chart of included patients.

Out of 1228 patients originally identified, 170 patients were included after the initial exclusion criteria (as shown in more detail in a previous paper [25]). After further examination on the additional exclusion criteria of this study, 125 patients were available for analysis (Fig. 2).

Baseline characteristics and surgical details did not differ with statistical significance between the two groups (Table 1).

**Table 1:** Baseline characteristics of included patients.

	Intravenous iron treatment			Patients with missing data (n)
	No	Yes	p-value	
n (%)	36	89		
Sex, Female, n (%)	18 (50)	37 (41.6)	0.39	0
Age in years, median (iqr)	73 (63–80)	75 (70–82)	0.07	0
Smoking status upon diagnosis, n (%)				
Nonsmoker	19 (57.6)	39 (45.9)		
Former smoker >8 weeks since smoking	7 (21.2)	31 (36.5)		
Active smoker	7 (21.2)	15 (17.6)	0.28	7
Alcohol consumption upon diagnosis (units/week), n (%)				
≤14	32 (91.4)	80 (93)		
>14	3 (8.6)	6 (7)	0.76	4
BMI, median (mean, sd)	25.41 (4.81)	26.56 (3.71)	0.15	0
ASA classification, n (%)				
ASA 1–2	22 (62.9)	50 (56.2)		
ASA 3–4	13 (37.1)	39 (43.2)	0.50	1
Tumor location, n (%)				
Right side^a^	25 (69.4)	68 (76.4)		
Left side	8 (22.2)	18 (20.2)		
Rectum	3 (8.2)	3 (3.4)	0.47	0
UICC-stage, n (%)				
UICC 1–2	20 (55.6)	54 (60.7)		
UICC 3–4	16 (44.4)	35 (39.3)	0.60	0
Hemoglobin, baseline (g/dl), median (iqr)	10.31 (7.17–11.60)	9.51 (7.90–10.80)	0.20	0
P-ferritin, baseline (μg/l), median (iqr)	15.5 (9–25.5)	13 (7–20)	0.34	0
TSAT baseline, median (iqr)	0.05 (0.03–0.09)	0.05 (0.03–0.08)	0.59	0
Conversion to open surgery, n (%)	3 (8.3)	5 (5.6)	0.57	0
Tumor perforation (intraoperative), n (%)	0	2	0.36	0
Radical primary surgery	31 (88.6)	81 (91)	0.68	1
dMMR	14 (38.9)	38 (42.7)	0.70	0

dMMR “Miss match repair deficiency”, TSAT “Transferrin saturation”.

a Including the transverse colon.

**Fig. 3:**
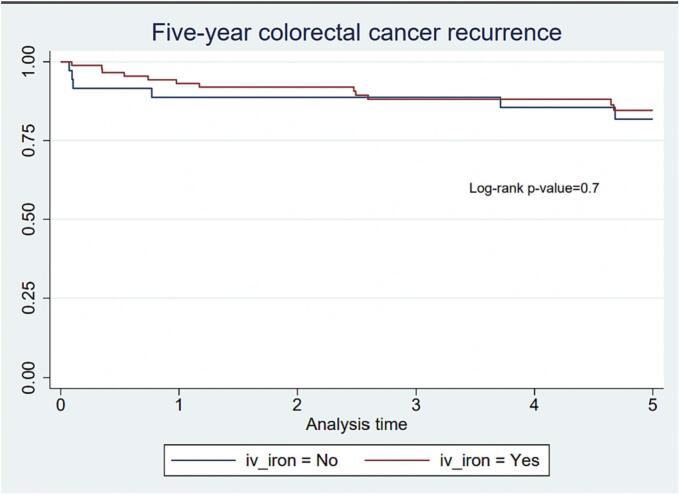
Kaplan Meier curve of time-to-recurrence comparing patients receiving preoperative intravenous iron with patients not receiving this treatment.

The overall five-year recurrence rate was 14 % (n = 18). 12 patients (13.5 %) in the group receiving i.v. iron and 6 patients (16.7 %) in the control group experienced a CRC recurrence. As presented with Kaplan-Meier curves this translated to an estimated five-year rate of non-recurrence of 85 % (0.74–0.91) in the i.v. iron treated group vs. 82 % (0.64–0.91) in the control group, non-significant difference (log-rank test p = .69) (Fig. 3). In i.v. iron treated patients, median follow-up time was 4.74 years (IQR 2.66–5) and 8 % (n = 7) were diagnosed with non-CRC cancer and 13.5 % (n = 12) died during follow-up. In the non-i.v. iron treated patients, median follow-up time was 5.00 years (IQR 3.74–5) and 8 % (n = 3) were diagnosed with non-CRC cancer and 2.8 % (n = 1) died during follow-up. There were no patients censored due to emigration.

Inspection of the Kaplan Meier curve (Fig. 3) suggests an association between i.v. iron treatment and recurrence rates during the first year of follow-up. Also, inspection of the log-log Kaplan-Meier curves did not clearly satisfy the proportional hazards assumption. We therefore performed a subsequent analysis splitting the follow-up time after one-year resulting in two separate periods, both supporting the proportional hazards assumption. Neither of those yielded statistically significant results on the associations between i.v. iron treatment and recurrence.

Table 2 shows the hazard ratios obtained when performing the multivariate analysis. No significant association between i.v. iron treatment and five-year CRC recurrence was found. Additionally, there was no significant correlation when examining the first year and the remaining four years individually.

**Table 2:** Multivariate competing risk analysis, estimating the association between intravenous iron treatment and five-year CRC recurrence.

I.v. iron vs no i.v. iron	Univariate hazard ratio (95 % ci)	Multivariate hazard ratio (95 % ci)^a^
CRC recurrence, overall	0.82 (0.31–2.18)	0.88 (0.31–2.51)
CRC recurrence, first year of follow-up^b^	0.58 (0.16–2.04)	0.57 (0.14–2.22)
CRC recurrence, second-fifth year of follow-up^b^	1.33 (0.27–6.58)	1.52 (0.30–7.80)

a Adjusted for UICC-stage, surgical radicality, gender, age, ASA group and miss match repair status.

b Follow-up is initiated at day 180 post-surgery.

## Discussion and conclusions

We found no association between preoperative i.v. iron treatment and five-year recurrence rates in colorectal cancer patients with iron deficiency anemia. To our knowledge, this is the first study on the long-term oncological associations of preoperative i.v. iron treatment in CRC patients, including only those with confirmed preoperative iron deficiency.

Multivariate regression found no statistically significant associations with recurrence. In the regression model, we adjusted for available variables theoretically affecting recurrence rates, including age, gender, UICC-stage, surgical radicality, comorbidity and miss match repair status. Our results are similar to the two available studies on i.v. iron treatment and CRC recurrence [21,22]. In addition to the theoretical direct effects of iron on tumor microenvironment and on immune system, preoperative iron treatment has been expected to lower red blood cell transfusion (RBCT) rates, and thereby improving long-term oncological outcome since perioperative RBCT are suggested to increase cancer recurrence [13]. However, as we have previously shown, and as found in two out of three randomized controlled trials preoperative i.v. iron does not as expected lower the perioperative RBCT rates [10,11,25,32]. Thus, current evidence suggests no beneficial nor any harmful long-term impact of i.v. iron treatment.

While currently no scientific evidence has documented a clinical short-term or long-term benefit of preoperative i.v. iron treatment in anemic CRC patients, a few studies now also have failed to find evidence supporting the speculated long-term detrimental effects of iron therapy. As i.v. iron maybe very successful in treating anemia in CRC patients, further research into possible beneficial consequences should be pursued [10]. We found in our cohort a significant proportion of patients not receiving preoperative i.v. iron as recommended by local guidelines. Since preoperative treatment seems to have no impact on the immediate surgical visit, in future, treating patients in the postoperative setting might prove easier to administer and with fewer patients not receiving treatments.

The strengths of the present study include the consecutive cohort and the data completeness of the included registries. The major limitations are the risk of selection bias together with the relatively small cohort available for analysis. It is possible that patients receiving i.v. iron differ systematically from patients not receiving this treatment, considering that local guidelines instructed clinicians to administer preoperative i.v. iron to all iron deficient anemic patients. We have previously addressed and explored this potential bias and found no systematic reasons for not receiving i.v. iron [25]. Also, as seen from Table 1, the two groups are comparable on baseline and disease specific parameters. There was however an unexpected and pronounced difference in long-term mortality-rates, which tended to be higher in the iron treated group. This could indicate that the i.v. iron treated patients were of poor general health compared to the control group.

Based on a consecutive cohort of 1228 CRC patients, after inclusion criteria were applied, we had 125 patients for recurrence analysis. This is a double-edged sword – by being very specific with defining the cohort it limits the final number of patients available for analysis. This entails the risk that smaller effects of i.v. iron treatment on recurrence than we would be able to identify with this study size might still exist.

This study included only patients with iron deficiency anemia and focused exclusively on recurrence. By this, the absent effect of iron treatment in non‑iron deficient patients does not confound the results and the possible oncological effects of iron treatment on recurrence is not blurred by inclusion of death as an outcome.

In conclusion, we found no association between preoperative i.v. iron treatment and the five-year cancer recurrence rate in iron deficient anemic CRC patients.

## Funding sources

This work was supported by grants from “The region of Southern Denmark”, “The 10.13039/501100006356University of Southern Denmark, SDU” and from Pharmacosmos A/S.

## Ethics approval

The need for informed consent was waivered by the Danish Patient Safety Authority (record-ID 3-3013-1497/1).

## CRediT authorship contribution statement

**Magnus Ploug:** Conceptualization, Methodology, Formal analysis, Writing – original draft, Funding acquisition. **Niels Qvist:** Conceptualization, Methodology, Writing – review & editing, Supervision. **Rasmus Kroijer:** Conceptualization, Methodology, Writing – review & editing, Supervision. **Torben Knudsen:** Conceptualization, Methodology, Writing – review & editing, Supervision.

## Declaration of competing interest

The study has partially been funded by Pharmacosmos A/S, manufacturer of intravenous iron products. Pharmacosmos A/S has not been involved in designing the study or analyzing the data. A drafted version on the manuscript has been available for Pharmacosmos A/S to comment on before submission. The authors were held by no obligations to incorporate these comments in the final manuscript. The authors MP, RK and TK have all received personal fees and travel expenses from Pharmacosmos A/S outside the submitted work. The authors have no further potential conflict of interest.

## Figures and Tables

**Table A.** 

Name of registry	Content relevant for this study	Time period of requested data
The Danish National Patient Register (NPR) [33]	ICD-10 (*International Classification of Diseases, 10th Revision)* codes on all patient contacts to hospitals in Denmark – both inpatients and outpatients. Information on all diagnoses registered on the patient as well as all in-hospital treatments.	2008 – end of study^a^
The Danish Civil Registration System (CRS) [34]	Dates of emigration or death	2013 - end of study^a^
Danish Cancer Registry (DCR) [35]	Records of all incidences of malignant neoplasms in Denmark	1978 - end of study^a^
Danish Pathology Registry (DNPR) [36]	Information on all pathology examination conducted in Denmark, including diagnoses based on the Danish SNOMED (Systemized Nomenclature of Medicine) codes	2013 - end of study^a^

a Final collection of data was done on Jan 31st, 2023.
